# Calcium signaling in the cochlea – Molecular mechanisms and physiopathological implications

**DOI:** 10.1186/1478-811X-10-20

**Published:** 2012-07-12

**Authors:** Federico Ceriani, Fabio Mammano

**Affiliations:** 1 Dipartimento di Fisica e Astronomia “G. Galilei”, Università di Padova, 35131, Padova, Italy; 2 Istituto Veneto di Medicina Molecolare, Fondazione per la Ricerca Biomedica Avanzata, 35129, Padova, Italy; 3 Istituto CNR di Neuroscienze, 35131, Padova, Italy; 4 VIMM, Via G. Orus 2, 35129, Padova, Italy

**Keywords:** Hearing, Inner ear, Transducer adaptation, Ca^2+^ channels, Cadherin 23, Protocadherin 15, Plasma-membrane Ca^2+^-ATPase, Prestin, Intracellular stores, Calcium release, Mitochondria, Ribbon synapse, Adenosine-5'-triphosphate, Inositol 1,4,5-trisphosphate, Connexin 26, Connexin 30, Deafness, Mouse models

## Abstract

Calcium ions (Ca^2+^) regulate numerous and diverse aspects of cochlear and vestibular physiology. This review focuses on the Ca^2+^ control of mechanotransduction and synaptic transmission in sensory hair cells, as well as on Ca^2+^ signalling in non-sensory cells of the developing cochlea.

## Review

### Introduction

The *cochlea* is the snail–shaped inner ear structure where auditory processing is initiated. Different regions of the cochlear *basilar membrane* vibrate at different sinusoidal frequencies due to variations in membrane thickness and width from the *base* (high frequency) to the *apex* (low frequency) of the cochlea. The basilar membrane supports a polarized sensory epithelium, the organ of Corti, which is responsible for *sound transduction*; it has the form of an epithelial ridge encompassing highly specialized sensory *inner hair cells* (IHCs) and *outer hair cells* (OHCs) characterized by a mechanosensory organelle composed of a stereociliary bundle protruding from the endolymphatic (apical) pole [1] (for schematic drawings of cochlear structures and cellular components, see e.g. Ref. [2]). All cells providing mechanical support to hair cells are designated as *supporting cells* and, in the cochlea, these are flanked by epithelial cells. In the following, supporting and epithelial cells of the sensory epithelium will be collectively designated as *cochlear non–sensory cells*. In the mature organ of Corti, the supporting cells include inner *phalangeal cells*, inner and outer *pillar cells*, outer *phalangeal cells* (also known as *Deiters’* cells), as well as *Hensen’s, Böttcher’s* and *Claudius’ cells*. In the lateral direction from the organ of Corti, the epithelium comprises *spiral prominence* cells and marginal cells of the *stria vascularis*. Tight junctions between neighboring cells prevent diffusion of proteins between the apical and basolateral domain of hair cells and supporting cells, and insulate endolymph, the unusual extracellular fluid low in Na^+^ and Ca^2+^ but rich in K^+^ that bathes the apical pole of epithelium, from perilymph, the normal extracellular fluid the bathes the basolateral membrane of the cells. In adult wild-type rats, endolymph Ca^2+^ concentrations have been reported as 20–30 μM [3]. Although the detatils of endolymphatic Ca^2+^ development are not known, it has been reported that a mature composition of endolymph in the mouse cochlea is reached around postnatal (P) day 8 (P8, where P0 indicates date of birth) [4]. The stria vascularis is responsible for exporting K^+^ to endolymph and generation of the *endocochlear potential* (reviewed in refs. [5-7]), an electrical potential difference between the endolymphatic and perilymphatic compartments of the cochlea, which in rodents appears around P5 and increases progressively to reach adult levels (in excess of +100 mV in mice) by P17 [8-11].

Both the endocochlear potential and the high endolymphatic [K^+^ are key factors for the *mechanotransduction process* performed by cochlear hair cells when mechanical stimuli are applied to their stereocilia bundle. Mechanotransduction relies on the large potential difference between the endolymph and the cytoplasm of IHCs and OHCs, which drives K^+^ through mechanically gated channels in the stereociliary bundle [12]. In adult hair cells, K^+^ influx through mechanotransduction channels evokes a receptor potential, i.e. a graded change of their resting membrane potential, *V*_*m*_. This analogue modulation is used by the IHC to activate the synaptic machinery present at its basal pole. By contrast, it is well established that OHCs use their receptor potential to fuel the local mechanical amplification process, driven by the motor protein *prestin*, which is required for the high sensitivity and sharp frequency selectivity of mammalian hearing (reviewed in Ref. [13]; see also [14,15]), however their sensory function is poorly understood.

During the period of time that precedes the acquisition of hearing, which in most rodents occurs around the second week after birth, the sensory epithelium is formed by the juxtaposition of the *greater epithelial ridge*, which gives rise to the IHCs and medial non–sensory cells, and the adjacent *lesser epithelial ridge*, which is thought to give rise to the OHCs and lateral non–sensory cells [16,17]. Hearing acquisition relies not only on the functional maturation of hair cells, but also on differentiation and proper organization of non–sensory cell networks that couple transfer of signaling, ion, and nutrient molecules through gap junction channels (reviewed in ref. [18]). The *epithelial* gap junction network forms around embryonic day 16 and connects all supporting cells in the organ of Corti as well as adjacent epithelial cells. A second network, named *connective tissue* gap junction network, starts to develop around birth and comprises interdental cells and fibrocytes in the spiral limbus, fibrocytes of the spiral ligament, basal and intermediate cells of the stria vascularis (reviewed in refs. [19,20]). In the so called *K*^*+*^*recycle* (or *recirculation*) *hypothesis* (reviewed in refs. [21-23]), the gap junction networks of the hearing cochlea are presumed to intervene during mechanotransduction, performing spatial buffering of the K^+^ released by the hair cells through K^+^ channels in their basolateral membrane.

Calcium ions (Ca^2+^) play numerous and fundamental roles in the inner ear. In the first part of this review, we focus on the aspects of sound transduction that are influenced by Ca^2+^, including mechanotransduction function and neurotransmitter release at the hair cell synapse. In the second part, we concentrate on Ca^2+^ signaling in the network of non-sensory cells in the developing cochlea.

#### Ca^2+^ at the hair cell endolymphatic pole

In the cochlea, the relative motion between the sensory cells and their overlaying structure, the tectorial membrane, causes the deflection of the hair bundle and the opening of mechanotransduction channels, one of the few ion channels not yet conclusively identified [24]. Stereocilia in the hair bundle are arranged in rows of graded height [25] and a fine extracellular filament, termed the tip link, connects the top of each stereocilium to the side of its taller neighbor, parallel to the bundle’s axis of mechanical sensitivity [26]. Tip-links are mechanically in series with a yet unidentified elastic element, termed *gating-spring*[26,27], which pulls on transduction channels and whose stiffness may be Ca^2+^-dependent [28]. It is thought that the hair cell receptor potential is caused by deflection of the hair bundle towards the tallest stereocilia, which increases the tension in the tip link causing the opening of mechanotransduction channels located at its bottom end [12]. Indeed, application of the Ca^2+^ chelator BAPTA to the hair bundle disrupts the tip links and abolishes mechanotransduction currents [29-31]. Cadherin 23 and protocadherin 15, respectively comprising 27 and 11 cadherin repeats, with Ca^2+^ binding sites in the interrepeat regions, interact at their N termini forming the upper (cadherin 23) and lower (protocadherin 15) part of tip links [32]. Furthermore, Molecular Dynamics simulations of the first two repeats of cadherin 23 suggest that Ca^2+^ binding at interrepeat sites is essential to determine cadherin 23 stiffness and folding strength [33]. A Ca^2+^ binding motif has also been identified at the N terminus of cadherin 23 [34], which could play an important role in the formation of the tip link and might also account for the disruptive effects of BAPTA [29,30]. Recent results in transgenic mice provide genetic evidence consistent with protocadherin 15 and cadherin 23 being part of the tip-link complex and necessary for normal mechanotransduction [35]. Further support to the tip-link model of transduction is derived from the evidence that mutations in genes encoding for cadherin 23 and protocadherin 15 have been associated with Usher syndrome type 1 and nonsyndromic hearing loss DFNB12 and DFNB23 [36-38].

Not only is Ca^2+^ essential to preserve the structure of the tip-links, but it also contributes to the mechanotransduction current. Based on early experiments performed under mM levels of extracellular Ca^2+^ concentration ([Ca^2+^_o_), it had been concluded that ~10% of the total mechanotransduction current was carried by Ca^2+^[39]. However, as previously mentioned, [Ca^2+^_o_ in endolymph is as low as a few tens of μM [3,40] and recent work indicates that the fraction of mechanotransduction current attributable to Ca^2+^ decrease in proportion to [Ca^2+^_o_; at endolympatic levels of [Ca^2+^_o_, it accounts for only the ~0.2% of the total mechanotransduction current [41]. In a standard experimental protocol, adaptation is measured by the decrease in mechanotransduction current, which occurs during a sustained deflection of the hair bundle [42]. Under these experimental conditions, adaptation shifts the relationship between the channel open probability (P_0_) and the bundle displacement (X) in the direction of the applied stimulus, canceling the effects due to sustained stimuli while maintaining the sensitivity to transient stimuli [43]. In this complex phenomenon, at least two phases, both Ca^2+^-dependent, can be distinguished: (i) fast adaptation, which occurs when Ca^2+^ enters transduction channels, then closes them within a few milliseconds or less; (ii) slow adaptation, which occurs with a time constant that spans a wide range of 10-50 ms depending on the type of hair cells studied (reviewed in [24]).

Fast adaptation is thought to be caused by the direct binding of Ca^2+^ to an intracellular site of the mechanotransduction channel or a closely associated subunit which closes the channel itself [28,44-47]. Slow adaptation, which has been studied extensively in vestibular hair cells, has been linked to activity of molecular motors [42], composed of unconventional myosin molecules [48-50], which interact in a Ca^2+^-dependent manner with actin filaments at the core of stereocilia [51]. The known stereocilia myosins that could affect adaptation in both IHCs and OHCs are myosin-Ic, VIIa, and IIIa [52-54]. Another unconventional myosin, XVa, which is required for normal growth of hair cell stereocilia, has been implicated in fast adaptation based on a study of P1-P4 shaker 2 mice (that have no functional myosin-XVa) [55]. This study indicates that: (i) Ca^2+^ sensitivity of the mechanotransduction channels and the fast adaptation require a structural environment that is dependent on this unconventional myosin; (ii) this environment is disrupted in IHCs of this mutant strain, but not in OHCs. However, available data indicate that myosin-XVa is present at the tips of wild-type stereocilia in both IHCs and OHCs [56,57]. Thus, to account for the different effects of myosin-XVa deficiency in OHCs and IHCs, it has been suggested that the loss of fast adaptation in IHCs of shaker 2 mice is associated with an unusual hair bundle architecture in these cells [55].

As for the mechanism of slow adaptation, in the classical scheme the motor complex is located in series with the transduction channel and its spring and is continuously trying to “climb up” the stereocilium, changing the position of the upper end of the tip link, thus increasing tension. Following a positive stimulus, the motor complex “slides down”, decreasing tension in the tip link and closing the channel [51]. However, analysis of the time course and pattern of myosin-Ic expression in IHC and OHC stereocilia [53,58] poses several challenges to the motor model of adaptation [24]. Furthermore, a motor complex located at the upper end of the tip link is hard to reconcile with the localization of the mechanotransduction channel, and thus of the site of Ca^2+^ entry, at the lower end of the tip link [12]. In order to resolve this conundrum, it has been proposed that Ca^2+^ entering through a transduction channel might affect the adaptation motor hooked up to the next tip link lower down the same stereocilium (for an explicative scheme, see Fig.5 of Ref. [51]). This implies that the tallest rows of stereocilia, which do not admit Ca^2+^ through mechanotransduction channels, are not likely to present Ca^2+^-dependent adaptation.

It is probably worth mentioning also that Ca^2+^ can influence mechanotransduction via cyclic adenosine monophosphate (cAMP), which has been shown to affect the response-displacement curve of the transducer [59]. This signaling pathway may involve cAMP production by Ca^2+^-calmodulin activated type I adenyl cyclase [60], cAMP-induced activation of protein kinase A and phosphorylation of the mechanotransduction channel or the myosin motor [43].

After entering the stereocilia through mechanotransduction channels, Ca^2+^ is rapidly bound by endogenous Ca^2+^ chelators, present at millimolar concentration [61], which restrict the distance Ca^2+^ diffuses to a few tens of nm from the mouth of the channel [39]. Also mitochondria, conspicuously concentrated in a band beneath the cuticular plate (the cytoskeletal anchor for the stereociliary bundle) [62,63], can act as large-capacity Ca^2+^ store [41]. In OHCs, the mitochondrial barrier may be bypassed by ATP-induced release of Ca^2+^ from a system of endoplasmic reticulum membranes located beneath the cuticular plate known as Hensen’s body [63].

Ca^2+^ is eventually exported back to endolymph by plasma membrane Ca^2+^-ATPase (PMCA) pumps, which are highly expressed in the hair bundle of vestibular and cochlear OHCs and, to a lesser extent, IHCs [64]. The stereociliary PMCA can be sufficiently active to elicit a substantial membrane current during transduction [65,66]. The pump isoform of the stereocilia is the PMCA2, encoded by the *ATP2b2* gene [67-70]. The extrusion task is performed by the *w/a* splicing isoform of PMCA2 [71,72]. Ablation of the *ATP2b2* gene causes deafness and balance disorders in mice [68], furthermore, various PMCA2 mutations have been linked to hereditary hearing loss in mice and humans. Some of the mutations described so far led to the truncation of the molecule and to its eventual disappearance from the stereocilia of the hair cell [68,70,73]. Three of the described mutations were instead point mutations that did not compromise the reading frame of the gene and were, thus, compatible with the expression of the full length PMCA2*w/a* variant of the pump; they all affected residues that are highly conserved in all PMCA isoforms across species and in other P-type pumps [69,74,75]. Recently, the *Tommy* mouse mutation was identified as a new PMCA2 pump mutant with progressive deafness from an ENU mutagenesis screen [76]. These mice show profound hearing impairment from P18, with significant differences in hearing thresholds between wild type and heterozygotes. Furthermore, immunofluorescence studies of the organ of Corti in homozygous Tommy mice showed a progressive degeneration of hair cells after P40 from the base of the cochlea (where high frequencies are detected) to its apex (low frequency region; see Introduction).

Due to the crucial role of Ca^2+^ at the endolymphatic pole of the hair cell for the performance of the mechanotransduction channel, a diminished Ca^2+^ removal from the stereocilia is expected to affect the mechanotransduction currents. Indeed, pharmacological blockade [41], as well as mutation or knock out of the PMCA2 pump [77] have been reported to shift the current-displacement (I-X) curve in the positive direction and to reduce its slope considerably. Moreover, the only cochlear PMCA2 exposed to endolymph is that of the stereocilia [64,78]. Thus if less Ca^2+^ is exported from the stereocilia , its concentration in the endolymph is expected to fall [78]. This may provide a clue as to why, in some cases, mutations in the gene of the PMCA2 pump potentiated the deafness phenotype induced by coexisting mutation of Cadherin 23 [77,79,80].

#### Ca^2+^ regulation of synaptic transmission

As mentioned in the introduction, mature hair cells respond to hair bundle deflection with graded changes in their membrane potential, which ultimately result in neurotransmitter release from the cell synaptic pole (see [81] for review). In contrast, before the onset of hearing, IHCs do not generate graded sound-driven receptor potentials but fire spontaneous Ca^2+^-driven action potentials [82-84]. These are prevented in mature IHCs by the expression of the rapidly activating large-conductance Ca^2+^-activated K^+^ current [85,86] and the negatively activating delayed rectifier I_K,n_, carried by KCNQ4 channels [83] (see [87] for a review). The IHCs synapses are already functional in the pre-hearing period [88], and glutamate release triggered by action potentials may be important for the refinement of the synaptic connections in the auditory pathway [89].

The hair cell synaptic machinery is unique in its genre, because of the special tasks it is required to accomplish. This is especially evident for the afferent synapse of cochlear IHCs, which must encode a wide range of external sound stimuli with the sub-millisecond temporal precision required for sound localization and phase locking; moreover, the constant presence of acoustic stimulation requires the prolonged maintenance of synaptic transmission [90]. The ability to produce both rapid and sustained neurotransmitter release is thought to be conferred to the hair cell synapse by the presence of the *synaptic ribbon*, a specialized electron-dense proteinaceous structure anchored at the synapse’s active zone, where synaptic vesicle exocytosis occurs [91]. This organelle, also found in retinal photoreceptor and bipolar cells (see [92] for a review), tethers ~100-400 glutamate-containing synaptic vesicles through thin filaments [93,94]. Some of these vesicles (~16-30) are kept in direct contact with the plasma membrane [95,96] and it has been suggested that the ribbon may be important for synchronous multi-vesicular release [96-99].

Vesicle exocytosis is triggered by the influx of Ca^2+^ through class-D L-type Ca^2+^ channels (Ca_V_1.3) clustered at each active zone [100-102] (~80 per active zone in mouse IHCs [103]), where, they operate in close proximity with Ca^2+^-activated K^+^ channels (BK channels) [85]. In mammalian IHCs and OHCs, the majority of the total Ca^2+^ current (>90%) is carried by Ca_V_1.3 channels [100,104]. It has been proposed that harmonin, a scaffolding protein that has been also implicated in mechanotrasduction at the level of the hair bundle [105,106], tags Ca_V_1.3 channels for ubiquitination and may thus constrain the number of presynaptic Ca_V_1.3 channels in IHCs [107]. The biophysical properties of these channels make them particularly suitable for the demands of synaptic transmission in these cells. First, Ca_V_1.3 channels activate at relatively hyperpolarized membrane potential, as negative as -70 mV in immature IHCs [100,108], indicating that they would be capable of generating both the spontaneous action potentials in immature IHCs and the fast synaptic response of mature hair cells [84]. Second, they activate very rapidly (~300-400 μs in gerbil basal IHCs) and show very little inactivation in mature hair cells [109,110], a characteristic that is required for sustained release. The exocytosis of individual fusion-competent vesicles is mediated by the stochastic gating of one or few Ca_V_1.3 channels located within a few nanometer from the release site and such “nanodomain control” of neurotransmitter has been proposed to permit temporally precise synaptic coding even for weak stimuli [103].

Because Ca_V_1.3 channels show strong Ca^2+^-dependent inactivation when studied in heterologous expression systems [111], it has been proposed that calmodulin-like Ca^2+^ binding protein (CaBP), which are expressed within the organ of Corti, may moderate the inactivation in cochlear IHCs by competing with calmodulin binding to the channel’s C-terminus [112,113]. Recent work has suggested that Rab3-interacting molecule-2 (RIM2) proteins may represent another possible molecular mechanism capable of inhibiting Ca^2+^- and voltage-dependent inactivation of Ca_V_1.3 channels in IHCs [114].

Similarly to stereociliary Ca^2+^, presynaptic Ca^2+^ domains are presumably spatiotemporally restricted by the presence of mobile, proteinaceous Ca^2+^ buffers calretinin, calbindin and parvalbumin, which have been found in a variety of cochlear and vestibular hair cells with concentration in the mM range [61,115]. However, discrepancies regarding both the amount and kinetic properties of such buffers in different hair cells suggest that their exact role and scope of function need to be analyzed further [116]. It has also been suggested that the restriction of the available presynaptic space due to the presence of the ribbon and its associated vesicles defines a small cytoplasmic volume where Ca^2+^ buffers are saturated, thus permitting fast and large Ca^2+^ rises near release sites beneath the synaptic ribbon [99].

The stimulus-secretion coupling between the inward Ca^2+^ current and transmitter release has been investigated by measuring the increase in the hair cell membrane capacitance (ΔC_m_) following depolarization-triggered Ca^2+^ entry [103,117-120]. In these studies, at least two kinetic components of exocytosis are commonly distinguished: a fast initial component, which saturates within a few milliseconds, and one or more slower components, triggered by prolonged (tens of ms to s in duration) depolarizing steps; for a summary of different studies on size and kinetics of synaptic release components in hair cells see [91]. The fast component is generally thought to represent the release of a ready releasable pool (RRP) of vesicles which might co-localize with Ca^2+^ channels [121-123]. However, data establishing a direct link between vesicle location and release pools are limited [91].

Transmitter release evoked by membrane depolarization over the physiological voltage range (between the resting potential and ~ -20mV [81]) shows a linear dependence on Ca^2+^ influx , at least in high frequency IHCs [103,118,120,124]. This linear relationship, which extends to the postsynaptic current [98,124], is believed to allow the synapse to respond efficiently to both small and large stimuli, thus broadening the hair cell’s dynamic range. Transmitter release shows a higher order (3^rd^-5^th^ power) Ca^2+^-dependance when the hair cell is depolarized to positive holding potentials [124] or when exocytosis is triggered by Ca^2+^ uncaging (7 μM to 110 μM) [117]. Recently, using real-time capacitance measurements to identify saturable pools of vesicles, a superlinear release component requiring recruitment of vesicles to release sites has been identified, leading to the suggestion that Ca^2+^-dependent vesicle trafficking is responsible for this movement, which is required for hair cell synapses to maintain high rates of sustained vesicle fusion [125].

The identification of the molecular composition of the synaptic machinery of the hair cell remains a major challenge. The hair cell synapse lacks the most common protein involved in exocytosis, for example complexins, synapsins and synaptophysins [126-128]; moreover, even though neuronal SNARE proteins are expressed in IHCs, they may not be required for vesicle fusion at the IHC ribbon synapse [129]. A major gap in our understanding of the components of the synaptic ribbon relates to the identification of the Ca^2+^ sensor. Synaptotagmins (Syt) I-II are the conventional Ca^2+^-sensing proteins at neuronal synapses [130], but their role at the hair cell ribbon synapse is debated. Though earlier studies suggested that Syt I-II were not present in mature IHCs [126], more recent work has shown that they are transiently expressed in the cochlea [131-133]. However these studies came to different conclusions about Syt I-II importance for IHC synaptic transmission, since some of them suggest their involvement (Syt1: [131,132], Syt II: [132]) while others exclude it (Syt1: [133]. Syt II: [131, 133]). The observation that otoferlin deficient mice (Otof^-/-^) are profoundly deaf [134], and show impaired synaptic development and lack of exocytosis [135] prompted the proposal that otoferlin is the major Ca^2+^ sensor of synaptic vesicle fusion in cochlear hair cells [135,136]. However, even though recent evidence has shown that otoferlin may be involved in synaptic vesicle replenishment [137], its role as the Ca^2+^ sensor for exocytosis remains indetermined. Indeed, otoferlin is not found in IHCs of a hypothyroid rat model, even though those IHCs exhibited Ca^2+^-dependent exocytois [132,138]. Moreover, another study showed that Ca^2+^-evoked exocytosis in the first postnatal days (P0-P4) is both otoferlin- and Syt- independent [131]. Finally, the transition from a nonlinear to a linear order of exocytotic Ca^2+^-dependance observed before and after the onset of hearing doesn’t correlate with the qualitatively similar distribution of otoferlin found in immature and mature IHCs [119,139], and seems to depend on another molecular factor, which has been recentely identified as Synaptotagmin IV, an unconventional synaptotagmin [132].

Besides Ca^2+^ influx through voltage-gated Ca^2+^ channels of the basolateral plasma membrane, two other mechanisms, both implicated in the efferent control of hair cell function [140,141], may promote an increase of intracellular free Ca^2+^ concentration ([Ca^2+^_i_) at the basal pole of the hair cell.

The first mechanism is Ca^2+^ entry through *α*9*α*10 nicotinic acetylcholine receptors (nAChR) [142,143], which activates, via calmodulin, a hyperpolarizing small conductance potassium current (SK, for review, see [144]). The hyperpolarizing SK current (i) is required for sustaining the action potential activity and modulating action potential frequency when activated by ACh in immature IHCs [145-147] and (ii) mediates fast Ca^2+^-dependent decrease of axial stiffness in OHCs [148,149].

The second (interrelated) mechanism is calcium-induced calcium release (CICR), an autocatalytic mechanism whereby [Ca^2+^_i_ elevation induces Ca^2+^ release from internal stores through channels such as inositol-1,4,5-trisphosphate (IP_3_) receptors (IP_3_Rs) or ryanodine receptors (RyRs) [150]. CICR has been investigated in mammalian IHCs [151], OHCs [149,152-156] as well as in vestibular hair cells [157]. In particular, in IHCs, Ca^2+^ release from intracellular store has been found to modulate the fast outward Ca^2+^ activated K^+^ current (BK) [86,158], thus suggesting that RyRs and BK channels are functionally coupled and act to suppress fast neurotransmission [158].

#### Ca^2+^ signalling in cochlear non-sensory cells

As mentioned above, cochlear non-sensory cells form vast syncytia coupled by gap junction channels that, in the mammalian cochlea, are formed primarily by connexin26 and connexin30 protein subunits [159,160], respectively encoded by DNFB1 genes *GJB2* and *GJB6* (reviewed in refs. [161,162]). The fact that DFNB1 is the most common form of inherited deafness in Caucasian populations highlights the importance of connexins for hearing (reviewed in ref. [163]). Connexin26 and connexin30 share 77% amino acid identity and may assemble to form heteromeric and heterotypic gap junction channels [164-166]. To date, that of a human connexin26 gap junction channel is the only structure resolved by X-ray diffraction (at 3.5 Å resolution) [167], whereas the structure of connexin30 channels has been recently inferred by a combination of homology modeling and molecular dynamics [168].

Mouse models confirmed that connexin26 and connexin30 are essential for auditory function, as well as survival and development of the organ of Corti [169-175]. Though instrumental, these animal models also revealed some critical gaps in our current understanding of the role played by connexins in the inner ear and the etiology of deafness due to absent or mutated connexins. Thus, deafness and lack of endocochlear potential in connexin30(−/−) mice correlate with: (i) disruption of the endothelial barrier of the capillaries supplying the stria vascularis before endocochlear potential onset; (ii) significant down–regulation of betaine–homocysteine S–methyltransferase and (iii) local increase in homocysteine, a known factor of endothelial dysfunction [176], with no obvious link to gap junction channel function. In the same vein, the hypothesis that connexin dysfunction impacts primarily on K^+^ recycle is challenged by the identification of connexin26 human recessive deafness mutants, e.g. V84L [177], that are capable of forming functional channels [178]. Studies performed in model cells indicate that connexin26 V84L mutant channels are as permeable to K^+^ as the wild type channels, whereas it is the transfer of the Ca^2+^–mobilizing second messenger IP_3_ (and possibly of other key metabolites too) that is impaired [179,180]. Therefore the permeability of connexin gap junction channels to metabolites [181,182], and not simply to small inorganic ions, is likely to play an important role in development, physiology and etiology of connexin−related hearing impairment. For an in depth analysis of several other difficulties met by the idea that K^+^ flux through the hair cells dominates the supply of K^+^ to the stria vascularis to form a closed K^+^ cycle see refs. [183,184].

While the exact function of connexins expressed by non–sensory cells of the inner ear remains unclear, it is important to mention that they also form unpaired connexons, i.e. non−junctional connexin hemichannels [185-187]. Experiments performed with a combination of genetic interference in four different mouse lines and ATP biosensors [188] apposed to cochlear non−sensory cells indicate that connexin26 and connexin30 protein subunits form functional hemichannels, which can be detected at the endolymphatic surface of the sensory epithelium with CELAb antibodies [189], and release ATP into endolymph under physiological conditions [190,191]. ATP release had been previously proposed on the ground of experiments in which mechanical stimulation was applied by gently pipetting (once per 3–4 s with a 20 μl pipette) a solution containing glass beads (with a diameter of 30-50 μm) over a cochlear explant for a 15-min period [192]. The binding of extracellular ATP to G–protein coupled P2Y_2_ and P2Y_4_ receptors, also expressed on the endolymphatic surface of the developing sensory epithelium, activates phospholipase–C dependent generation of IP_3_[179,193,194]. While gap junction channels allow IP_3_ diffusion through these coupled cells, IP_3_ binding to its receptors (IP_3_R) promotes Ca^2+^ release from the endoplasmic reticulum raising the cytosolic free Ca^2+^ concentration ([Ca^2+^_i_). The probability of connexin hemichannel opening is a bell–shaped function of the [Ca^2+^_i_, peaking at ~500 nM [195]. This is a key feature that enables the propagation of Ca^2+^ signals as regenerative and coordinated intercellular Ca^2+^ waves, with peak amplitude of ~500 nM, sustained by ATP–induced ATP–release [2,179,190,191,193,194]. Mitochondria function as spatial Ca^2+^ buffers and play a significant role in regulating the spatio–temporal properties of these intercellular Ca^2+^ waves [196]. This was demonstrated by blocking mitochondrial Ca^2+^ uptake by dissipating the mitochondrial membrane potential using the protonophore carbonyl cyanide m–chlorophenylhydrazone (CCCP) and oligomycin, an inhibitor of oxidative phosphorylation, or using Ru360, an inhibitor of the mitochondrial Ca^2+^ uniporter, which enhanced the peak amplitude and duration of ATP–induced transients. The numerous roles played by extracellular ATP in the adult cochlea are reviewed in ref. [197]; the rest of this article focuses on some critical signalling events that occur during maturation of cochlear tissue.

Rhythms are ubiquitous at all levels of biological organization. At the cellular level, they involve biochemical oscillations that modulate the concentration of key metabolic substrates and second messengers. Among these, rhythmic variations in the [Ca^2+^_i_ have been found in a variety of cells and shown to arise spontaneously or after stimulation by hormones or neurotrasmitters. In non–sensory cells of the lesser epithelial ridge, ATP–dependent [Ca^2+^_i_ oscillations occur (i) as consequence of intercellular Ca^2+^ wave propagation, (ii) sustained ATP delivery in the submicromolar range or (iii) during pharmacological inhibition of ectonucleotidases, a manipulation which highlights the tonic release of ATP from these cells [190] and their sensitivity to ATP degradation by ectonucleotidases [198]. In rat cochlear explants [199,200], as well as in mouse organotypic cochlear cultures [175], [Ca^2+^_i_ transients due to release of ATP in rhythmic bursts have been reported also for a class of non–sensory cells of the greater epithelial ridge (first described by Kölliker) which transiently populate the sensory epithelium from spiral limbus to IHC [201,202]. These periodic Ca^2+^ signals can be blocked by apyrase, as shown for the propagation of intercellular Ca^2+^ waves in the lesser epithelial ridge. Furthermore, the frequency of spontaneous [Ca^2+^_i_ transients is significantly decreased by purinergic receptor antagonists PPADS (50 μM) and suramin (150 μM), the gap junction channel inhibitor carbenoxolone (100 μM) as well as flufenamic acid (50 μM), a bona–fide inhibitor of connexin hemichannels. Both the propagation range of intercellular Ca^2+^ waves in the lesser epithelial ridge and the frequency of spontaneous [Ca^2+^_i_ transient in the greater epithelial ridge increase when the extracellular free Ca^2+^ concentration ([Ca^2+^_o_) is decreased [190,199,200], and this manipulation is known to increase the open probability of connexin hemichannels [203-206]. Finally, focal UV photolysis of a caged intracellular IP_3_ precursor in the greater epithelial ridge evokes Ca^2+^ transients similar to those that arise spontaneously in this region [191]. Thus it seems reasonable to hypothesize that release of ATP through connexin hemichannels activates similar IP_3_ receptor–dependent signal transduction cascades in non–sensory cells of the lesser and the greater epithelial ridge.

These findings are particularly interesting if viewed from the perspective that connexin dysfunction may ensue in a deafness phenotype through a bidirectional link to impaired ATP–dependent Ca^2+^ signaling in the developing cochlea. This tenet is exemplified by a study of hearing loss based on the substitution of an evolutionarily conserved threonine by a methionine residue at position 5 near the N–terminus of connexin30 (connexin30 T5M) [207]. In connexin30^T5M/T5M^ knock in mice, obtained by homologous recombination in mouse embryonic stem cells, expression of the mutated connexin30 T5M protein is under the control of the endogenous connexin30 promoter [175]. When probed by auditory brainstem recordings, connexin30^T5M/T5M^ mice exhibit a mild, but significant increase in their hearing thresholds of about 15 dB at all frequencies. Western blot analysis of adult inner ear tissue shows significantly down–regulated expression levels of connexin26 and connexin30. In the developing cochlea, electrical coupling, probed by dual patch–clamp recordings, is normal; however, transfer of the fluorescent tracer calcein between cochlear non–sensory cells is reduced, as is the intercellular Ca^2+^ signalling due to spontaneous ATP release from connexin hemichannels [175]. Previous studies had noted that ATP–dependent Ca^2+^ oscillations in non–sensory cells of the cochlear feed–back on connexin expression and participate in the coordinated regulation of connexin26 and connexin30 through NF–kB [208,209] (nuclear factor kappa–light–chain–enhancer of activated B cells). Of notice, these articles also show that gene delivery with recombinant *bovine adeno associated virus* (BAAV) vectors restores connexin expression and rescue intercellular coupling and Ca^2+^ signaling in cochlear organotypic cultures from mice with defective expression of connexin26 and connexin30 [208,209]. A widely held hypothesis is that information is encoded mainly by the frequency of [Ca^2+^_i_ oscillations [210,211], however, a possible role of amplitudes and duration in signal transduction has been discussed [212,213]. It has also been argued that amplitude modulation and frequency modulation differentially regulate distinct targets [214]. Note that NF–kB is just one of the several Ca^2+^–dependent transcription factors used by non–excitable cells [215], thus future research will undoubtedly uncover more links between these molecular actors, hearing acquisition, and its failure due connexin dysfunction.

### Conclusions

The investigation of Ca^2+^ signaling in the context of the inner ear is a rapidly growing and accelerating field of research. Our knowledge about the molecular details is rapidly growing with new gene products identified as key players of mechanotransduction, while more and more molecules have been added to the list of those involved in synaptic transmission. Finally the interconnectivity of connexin expression and signaling pathways in the developing cochlea constitutes a key feature of the vast non-sensory cell syncitia which starts to emerge as fundamental part of the intricate machinery that fosters the acquisition of hearing. Despite some impressive proceedings in recent years, we are surely not running out of open questions.

## Competing interests

The authors declare no competing interests.

## Authors’ contributions

FM and FC wrote the paper. Both authors read and approved the final manuscript.
